# Modulation of Autophagy for Controlling Immunity

**DOI:** 10.3390/cells8020138

**Published:** 2019-02-09

**Authors:** Young Jin Jang, Jae Hwan Kim, Sanguine Byun

**Affiliations:** 1 Research Group of Natural Materials and Metabolism, Korea Food Research Institute, Wanjugun 55365, Korea; jyj616@kfri.re.kr; 2 Department of Agricultural Biotechnology, Seoul National University, Seoul 08826, Korea; kjhh720@snu.ac.kr; 3 Division of Bioengineering, Incheon National University, Incheon 22012, Korea

**Keywords:** autophagy, innate immunity, adaptive immunity, modulators

## Abstract

Autophagy is an essential process that maintains physiological homeostasis by promoting the transfer of cytoplasmic constituents to autophagolysosomes for degradation. In immune cells, the autophagy pathway plays an additional role in facilitating proper immunological functions. Specifically, the autophagy pathway can participate in controlling key steps in innate and adaptive immunity. Accordingly, alterations in autophagy have been linked to inflammatory diseases and defective immune responses against pathogens. In this review, we discuss the various roles of autophagy signaling in coordinating immune responses and how these activities are connected to pathological conditions. We highlight the therapeutic potential of autophagy modulators that can impact immune responses and the mechanisms of action responsible.

## 1. Introduction

The term autophagy is derived from the Greek words “auto” meaning self and “phagein” meaning to eat [1]. Autophagy is a highly conserved process in eukaryotes through which cell components are degraded to optimize activity and maintain viability, often in response to nutrient limitation [2]. The process is important for routine housekeeping needs, but also plays critical roles in various other biological processes at the cellular and physiological levels [3]. Autophagy also provides a critical line of defense against invading intracellular pathogens including viruses, bacteria, and protozoa, and can regulate immune cell responses [4,5]. In this review, we will describe recent discoveries concerning autophagy, as well as the effects of bioactive compounds that can influence such signaling during immune responses to pathogen attack, inflammation control, and the modulation of adaptive immunity through antigen presentation.

## 2. Autophagy Signaling

Autophagy helps to maintain cell homeostasis and is initiated when undesirable environmental cues are detected, such as nutrient starvation or physical and chemical stresses [5]. Complex mediators are involved in the autophagy process, including mammalian target of rapamycin complex 1 (mTORC1), AMP-activated protein kinase (AMPK), ULK1 (a serine-threonine protein kinase), the class III phosphatidylinositol-3-phosphate kinase (PI3K) complex, mammalian homolog of autophagy related proteins 8 (ATG8), and other factors that mediate the formation of autophagosomes and their fusion with intracellular lysosomes [4]. These functional molecular networks are activated upon detection of an appropriate stress signal and are regulated in a highly coordinated manner [6]. Under nutrient-rich conditions, mTORC1 suppresses ULK1 activity via phosphorylation, while a loss of mTORC1 activity during stress or nutrient starvation releases ULK1, initiating autophagy signaling [2]. Activated ULK1 promotes autophagy by subsequently phosphorylating Beclin 1 and activating the VPS34 lipid kinase. The class III phosphatidylinositol-3-phosphate kinase family is comprised of several lipid kinases that phosphorylate phosphatidylinositol at the hydroxyl group at position 3 of the inositol ring, producing phosphatidylinositol-3-phosphate (PtdIns(3)P) [3]. The PI3K complex, which is composed of VPS34 (catalytic subunit), Beclin 1, and ATG14L (ATG14 like protein), can then translocate to the endoplasmic reticulum (ER) following activation of the autophagy pathway [3]. PtdIns(3)P produced by the PI3K complex recruit double FYVE(Fab1p/YOTB/Vac1p/EEA1)-containing protein 1 (DFCP1) and WD repeat domain phosphoinositide-interacting (WIPI) family proteins to the ER [4]. DFCP1 promotes the formation of omegasomes (ER-associated Ω-like structures) and WIPI2, which colocalizes with DFCP1, accelerates the development of the omegasome into the autophagosome [7]. WIPI2 directly binds with ATG16L and recruits the ATG12–ATG5–ATG16L complex to the autophagosome formation site [8]. The ATG12–ATG5–ATG16L complex then promotes the addition of phosphatidylethanolamine (PE) to the carboxyl terminus of the mammalian paralogues of ATG8 (hereafter referred to as LC3): LC3A, LC3B, LC3C, γ-aminobutyric acid receptor-associated protein (GABARAP), GABARAP-like 1 (GABARAPL1), and GABARAPL2 [1]. The lipidation of LC3 paralogues leads to the completion of autophagosome formation [9]. For this reason, LC3 is used as a preferred marker for microscopic detection of isolation membranes and autophagosomes, while phosphatidylethanolamine-conjugated LC3 (LC3-II) is used as a marker of autophagic activity [3]. After fusing with a lysosome, the autophagosome matures into an autolysosome, leading to the degradation and digestion of its contents and inner membrane by lysosomal hydrolases [10].

## 3. Pathogen Degradation: Xenophagy

Autophagy was initially thought to be a non-selective degradation process, but it is now believed that autophagosomes can degrade substrates and intracellular pathogens in a targeted manner in a process referred to as xenophagy [3]. Selective autophagy can be classified into mitophagy (degradation of damaged mitochondria), pexophagy (peroxisomes), lipophagy (lipid droplets), glycophagy (glycogen), ribophagy (ribosomes), ER-phagy (ER), and xenophagy (intracellular pathogens) [11]. Xenophagy is defined as a selective autophagic process against pathogens and other non-host entities [12]. Xenophagy requires all of the molecular machinery involved in classical autophagy, and the selective degradation process is thought to be possible due to pattern recognition receptors (PRRs) and the marking of intracellular pathogens by cellular ubiquitinases [5]. The host protein ubiquilin 1 (UBQLN1) recognizes *Mycobacterium tuberculosis* and recruits autophagy machinery to induce xenophagic clearance of the invading bacterium [13]. Similarly, *Helicobactor pylori* can be degraded by xenophagy through ATG16L1 in gastric epithelial cells [14]. It has been consistently reported that some pathogens manage to survive intracellularly due to their ability to evade the host cell’s xenophagic response. For example, *Shigella flexneri* can escape xenophagy after invading the cell by secreting the protein IcsB, which interferes with the autophagic host defense system [15].

The immune system senses exogenous pathogens or endogenous stress via specialized PRR machinery that includes toll-like receptors (TLRs), sequestosome 1 (SQSTM1)-like receptors (SLRs), nucleotide oligomerization domain (NOD)-like receptors (NLRs), retinoic acid-inducible gene-I (RIG-I)-like receptors (RLRs), and absence in melanoma 2(AIM2)-like receptors (ALRs) [16]. PRRs recognize pathogen-associated molecular patterns (PAMPs) and damage-associated molecular patterns (DAMPs), and in turn activate autophagy [17]. It has been reported that at the site of bacterial entryNOD1 and NOD2, the founding members of the NLR family, can sense invasive bacteria and induce xenophagy by recruiting ATG16L1 [18]. SLRs such as p62, neighbor of BRCA1 gene 1(NBR1), and optineurin serve as adaptors between the ubiquitin tags on microbial targets (as well as other endogenous targets) and ATG8/LC3 [5], connecting the autophagic cargo to nascent autophagosomes [19]. The importance of SLRs has been studied extensively in xenophagy, with knockdown of p62 in macrophages shown to improve the survival of *M. tuberculosis* in the host cell [20]. In addition, xenophagy of ubiquitin-coated cytosolic *Salmonella enterica* is enhanced by the phosphorylation of optineurin, suggesting an important role for these adaptors in xenophagy [21]. Because each SLR exhibits varying affinity towards the different ubiquitin chains, non-ubiquitinated proteins, and Atg8 paralogues, SLRs in turn vary in their specificity for invading pathogens [5]. 

Viral replication and infection-induced cell death can also be attenuated by autophagy [22]. The autophagy protein Beclin 1 reduces Sindbis virus-induced apoptosis of brain cells and lethality by encephalitis in mice [23]. Upon respiratory syncytial virus infection, Beclin 1 in dendritic cells plays a critical role in antiviral adaptive immune responses by participating in MHC class II expression and innate cytokine production [24]. The Beclin 1 and ATG genes appear to be highly conserved throughout evolution and also play roles in pathogen responses in plants, being found to restrict programmed cell death (a form of host defense) to the tobacco mosaic virus (TMV) infection site [25]. Beclin 1 is a Bcl-2 anti-apoptotic gene-interacting protein that plays diverse roles in antiviral host defense [23]. p62 recognizes Sindbis virus capsid protein and delivers it to the autophagosome, demonstrating that autophagy is also capable of targeting individual viral capsids for degradation [26]. Knockdown of p62 or other autophagy genes has been shown to increase viral capsid accumulation and accelerate virus-induced cell death [26]. NBR1 also binds viral capsid proteins and particles of cauliflower mosaic virus (CaMV), thereby modulating their degradation by autophagy [27].

## 4. The Proviral Role of Autophagy

Although autophagy activation frequently serves as a defense mechanism against viral infection, many viruses have evolved to utilize the autophagy machinery for promoting their infection and replication [28]. Autophagy proteins such as ATG5 and Beclin 1 play a critical role in Japanese encephalitis virus replication [29]. Also, hepatitis C virus utilizes autophagy for the translation of its RNA as well as for the initiation of viral replication [30]. Formation of the autophagic membrane induced by hepatitis C virus infection can be used as a membrane compartment for the replication of viral RNA [28]. In addition, many viruses including Coxsackie B, Epstein–Barr virus, varicella-zoster virus, human papillomavirus 16, and simian virus 40 have been reported to activate autophagy to enhance viral infection [28,31]. Recently, it has been identified that the autophagic pathway is critical for maternal–fetal transmission of the Zika virus, suggesting that inhibition of autophagy could be a therapeutic approach to attenuate Zika virus infection and transmission [32]. 

Extensive studies on human immunodeficiency virus type 1 (HIV-1) further demonstrate the complex relationship between autophagy and antiviral immunity. It has been reported that autophagy is required for the replication of HIV-1, but autophagy can also suppress HIV-1 infection. In the early stage of primary infection, HIV promotes autophagy to maximize virion production. However, HIV suppresses the proteolytic and degradative late stages of autophagy to avoid the antiviral effect [33]. While suppressing autophagy factors such as ATG16 and ATG5 by gene silencing led to a decrease in HIV-1 replication [34], activation of autophagy has been implicated as a mean to restrict HIV-1 infection in T lymphocytes by selectively degrading a HIV-1 protein essential for viral transcription and virion production [35]. Hence, the role of autophagy on HIV replication differs depending on cell type (e.g., macrophage, T cell) and context. Similar contradicting results with mouse hepatitis virus have been found in a cell-type dependent manner. Replication of mouse hepatitis virus is decreased in ATG5−/− embryonic stem cells, but its replication was not suppressed in mouse embryonic fibroblasts and bone marrow derived macrophages in the absence of ATG5 [22,36]. Collectively, these reports suggest that autophagy can have both pro- and antiviral roles for the virus. Also, the complicated relationship between autophagy and virus pathogenesis appears to have a cell- and circumstance-specific manner, implying that understanding the function of autophagy in regulating viral infection should be carefully considered based on the type of virus and cells involved.

## 5. Autophagy and Innate Immune Systems: Regulation of Inflammation

The innate immune system promotes inflammation via the secretion of inflammatory mediators, such as type I interferon (IFN) and cytokines to fight microbial infection [3]. Autophagy is directly linked to PRR-mediated type I IFN signaling, and PAMPs are recognized by TLR7 following transport into the lysosome by autophagy-related processes [37]. This enables the production of IFN-α by plasmacytoid dendritic cells [37]. Although inflammation is essential for host defense, excessive inflammation can cause inflammatory diseases, including septic shock, allergies, and metabolic disorders [3]. Autophagy is intricately involved in the control of inflammation and essential for the correct functioning of the innate immune system [3]. Rubicon (RUN domain and cysteine-rich domain containing, Beclin 1-interacting protein), a major regulator of autophagy, is also a physiological feedback inhibitor of CARD9–BCL10–MALT1 signaling complexes, which can terminate PRR-induced cytokine production to prevent unchecked proinflammatory responses [38].

Autophagy directly regulates the secretion of inflammatory cytokines by inhibiting the inflammasome pathway [5]. Inflammasomes are cytoplasmic complexes that recognize microbial products, and are responsible for upregulating the secretion of inflammatory cytokines such as interleukin 1β (IL-1β) and IL-18 for host defense [39]. Because aberrant inflammasome activation can cause excessive inflammation and lead to severe tissue damage, the tight regulation of inflammasomes is essential for balanced immune responses [3,40]. The inflammasome is comprised of pro-caspase-1, the adaptor protein ASC, and a sensor protein from either the NLR (nucleotide-oligomerization domain (NOD) and leucinerich-repeat-containing) family or from the PYHIN (pyrin domain (PYD) and hematopoietic interferon-inducible nuclear (HIN) domain-containing) family (such as absent in melanoma 2 (AIM2) and IFNγ inducible protein 16(IFI16)) [1,40]. The activation of inflammasomes promotes the proteolytic maturation of interleukin 1β (IL-1β) and the related cytokine IL-18 by caspase-1. NLRP3 is a well-characterized PRR of the NLR family [3], and mitochondrial dysfunction can trigger inflammasome activation by NLRP3 [41]. PAMPs and DAMPs cause mitochondrial damage and the release of reactive oxygen species (ROS), leading to NLRP3 inflammasome activation and the production of IL-1β and IL-18 [42].

Depletion of the autophagic proteins LC3B and Beclin 1 promotes NLRP3-dependent inflammation via the accumulation of dysfunctional mitochondria and ROS [43]. Autophagic degradation of the mitochondria suppresses the activation of NLRP3 inflammasome and the production of IL-18 in a ULK1-dependent manner during virus infection [44]. Autophagy mediators also recognize ubiquitinated ASC and induce the selective degradation of inflammasomes, thereby inhibiting the production of IL-1β and IL-18 [3]. Autophagy controls the production of IL-1β by targeting pro-IL-1β for lysosomal degradation in addition to regulating the activation of NLRP3 inflammasomes [45]. 

Autophagy also regulates other pro-inflammatory signaling factors in addition to the inflammasome [1], degrading BCL-10 to reduce nuclear factor-κB (NF-κB) activation in antigen-activated T cells [46]. NF-κB signaling can also be inhibited by autophagy via p47, an essential factor in Golgi membrane fusion [47]. p47 induces the lysosomal degradation of polyubiquitinated NF-κB essential modulator [47], while murine cytomegalovirus protein M45 suppresses the inflammatory cascade by targeting NF-κB essential modulator to autophagosomes for degradation, a process that leads to the suppression of viral proliferation during infection [48].

## 6. Autophagy in the Adaptive Immune System: Regulation of Antigen Presentation

While the innate immune system senses pathogens via PRRs, the adaptive immune system recognizes processed peptides from pathogen- or tumor-associated proteins following their presentation on major histocompatibility complex (MHC) molecules [3]. Autophagy plays a crucial role in the presentation of MHC class I and II molecules for recognition by CD8+ and CD4+ T cells, respectively [49,50]. Extracellular antigens captured by antigen-presenting cells (APCs) are delivered to autophagosomes to enable the generation of immunogenic peptides prior to loading onto MHC class II molecules for presentation to CD4+ T cells [5,51]. Autophagy also promotes MHC class II presentation of peptides from intracellular source proteins [52]. Dendritic cells from patients with Crohn’s disease expressing NOD2 and ATG16L1 risk variants are defective in autophagy induction and MHC class II antigen presentation [53]. In line with this, rapamycin-induced autophagy enhances the presentation of mycobacterial antigens in macrophages and the CD4+ T cell response [54]. 

Intracellular antigens captured by autophagosomes can be degraded by amphisomes (which arise from the fusion of autophagosomes with endosomes) and loaded onto MHC class I molecules for presentation to CD8+ T cells [5]. Pathogens such as the herpes simplex virus type 1 (HSV-1) can initiate this process, triggering the processing and presentation of endogenous viral antigens on MHC class I molecules [55]. In addition, antigen-presenting cells, such as dendritic cells, can process extracellular antigens for MHC class I presentation by cross-presentation, a process that is also dependent on autophagy [56,57].

## 7. Autophagy in Immune Cell Function

Autophagy is broadly implicated in the survival, development, and maturation of immune cells. Beclin 1-deficient CD4+ T cells are prone to apoptosis after activation [58]. Deletion of ATG5 in T lymphocytes results in survival defects and proliferation issues caused by the accumulation of damaged and aging mitochondria [59]. The number of naive T cells is significantly lower in the absence of mitophagy, which serves as the autophagic clearance process for damaged mitochondria [3]. Autophagy induction via the inhibition of p38 in senescent CD8+ T cells increases their proliferation, telomerase activity, and mitochondrial biogenesis [60], while deletion of ATG5 in B lymphocytes negatively impacts their maturation and survival [61]. Moreover, autophagy is also essential for effector CD8+ T cell survival and memory formation, as CD8+ T cells lacking ATG7 exhibit cell-intrinsic defects which prevent their development into long-term memory cells [62].

## 8. Modulators of Autophagy for Immune Control

### 8.1. Spermine

Spermine is a natural polyamine that can be found in pumpkin, cheese, and most meat products, including pork, chicken, and turkey [63]. It has been reported to induce autophagy via histone deacetylation and p53 activation in cancer cells [64], and can elicit the upregulation of Beclin 1, LC3-I, and LC3-II, as well as the inhibition of mTOR in an ischemia/reperfusion injury (IRI) model [65,66]. Several studies have focused on the diverse immune-modulatory functions of spermine [67,68]. In a piglet model, spermine supplements were shown to alleviate the inflammatory response as evidenced by the downregulation of IL-1β, IL-2, IL-6, tumor necrosis factor-α (TNF-α), and IFN-γ levels in serum [67]. Spermine has been also implicated in other anti-inflammatory effects via ATG5-dependent autophagy in a C57BL/6J mouse model [68]. In a mouse model of liver injury induced by thioacetamide, spermine markedly suppressed M1 polarization via the suppression of *IL-1β* and *iNOS* gene induction, whereas in liver-resident macrophages (Kupffer cells) M2 polarization was promoted through the upregulation of *Arg-1* and *Mrc-1* gene induction. Simultaneously, spermine induced autophagy concomitant with an increase in LC3B-II levels and ATG5 protein expression and a decrease in p62 protein expression in thioacetamide-treated Kupffer cells. The upregulation of ATG5 in Kupffer cells treated with thioacetamide and spermine suggests that spermine-induced Kupffer cell autophagy is dependent on ATG5. Furthermore, intervention with ATG5 knockdown demonstrated that spermine-induced Kupffer cell autophagy could be eliminated with the restoration of *IL-1β* and *iNOS* (M1 markers) and *Arg-1* and *Mrc-1* (M2 markers) gene induction. These results demonstrate that spermine may attenuate thioacetamide-induced acute liver injury by enhancing autophagy in Kupffer cells in an ATG5-dependent manner.

### 8.2. Spermidine

Spermidine is another natural polyamine abundant in soybean, green peas, corn, chicken liver, shellfish, and blue cheese [63]. The compound has been observed to enhance longevity in yeast, flies, worms, and mice, with the major mechanism responsible thought to be an autophagy-inducing effect that occurs in an mTOR-independent manner [69,70,71]. According to recent animal studies, spermidine also appears to improve the immune response to infection [72] by counteracting the formation of defective CD8+ T cells that typically increase with aging. Interestingly, mice lacking the autophagy gene *Atg7* have also shown impairment of CD8+ T cell formation, which appears similar to aged immunity. Old mice have shown slower CD8+ T cell response compared to that of young mice during influenza vaccination. Contrastingly, CD8+ T cell response against influenza has been significantly amplified in old mice treated with spermidine, and autophagy level has also been upregulated in Jurkat cells treated with spermidine. Furthermore, CD8+ T cells from Atg7−/− mice treated with spermidine have not exhibited a response against influenza due to the absence of autophagy. Although mTOR has been shown to boost both the quantity and quality of specific CD8+ T cells responding to viral challenge [73], spermidine might not be dependent on the mTOR pathway [72].

### 8.3. Resveratrol

Resveratrol is a stilbenoid that is enriched in red wine and grapes and has been extensively investigated for its varied effects on human health. These include autophagy regulation in rheumatoid arthritis [74], osteoarthritis [75], hepatic steatosis [76,77], neuroprotection [78,79], cancer [80,81,82], and various other disease models [83,84,85,86]. Several studies have focused on the immuno-regulatory function of resveratrol, which can occur via the regulation of autophagy. In restraint-stressed mice, resveratrol ameliorates the apoptotic death of macrophages and enhanced stress-induced autophagy in mouse peritoneal macrophages via an increase in SIRT3 expression and phosphorylation of AMPK. Following this, resveratrol treatment was shown to rescue macrophages from apoptosis and activate the SIRT3–AMPK–autophagy positive feedback loop to prevent the generation of mitochondrial-reactive oxygen species (ROS) [87]. In another restraint-stressed mouse model study, resveratrol treatment improved splenic damage induced by restraint, which involved reductions in splenocyte and CD4+ T-cell numbers. Resveratrol treatment reversed the reduction of SIRT3 expression and increased Beclin 1 expression, as well as the converting LC3-I to LC3-II in splenocytes [88]. Resveratrol has been also reported to induce autophagy in a peritoneal inflammatory injury model. One study found that enhanced autophagy by resveratrol may prevent human peritoneal mesothelial cells from ROS-mediated NLRP3 inflammatory injury, and that resveratrol induced autophagy through AMPK activation in the SV40-immortalized human peritoneal mesothelial cell line [89]. In addition, resveratrol has been observed to enhance autophagy for the alleviation of vascular endothelial inflammation in an atherosclerosis model [90], protecting HUVECs from inflammation induced by TNF-α. To identify the mechanism responsible for reduced endothelial inflammation by resveratrol, gene silencing was used to show that resveratrol triggered autophagy via the cAMP–PRKA–AMPK–SIRT signaling pathway. Resveratrol is a well-known phytochemical and one of its prime targets is the SIRT–AMPK pathway. As AMPK plays a key role in autophagy, it appears that resveratrol maybe a useful modulator for controlling autophagy-mediated immune responses in diverse settings. 

### 8.4. Artesunate

Artesunate is an artemisinin drug that has been extensively investigated for its application as a malaria treatment [91,92,93]. In recent years, studies have demonstrated that artesunate is a promising therapeutic candidate for applications in arthritis [94,95], atherosclerosis [96], neurological disorders [97], and cancer [98,99,100,101]. Some reports have suggested that autophagy control is responsible for the therapeutic effect elicited by artesunate. In one study, autophagy activation following artesunate treatment was suggested to be responsible for protective effects against hypoxia-induced hippocampal neuronal death and brain injury in an ischemic cerebral infarction model [97]. In addition, artesunate was reported to suppress proliferation of fibroblasts via the activation of autophagy during epidural fibrosis. [102]. In contrast, another study found that artesunate inhibited autophagy in macrophages [103]. Artesunate reduced the production of pro-inflammatory cytokines, TNF-α, and IL-6 in RAW 264.7 cells, mouse bone marrow-derived macrophages, and peritoneal macrophages. The anti-inflammatory effects of artesunate were closely linked with its ability to inhibit lipopolysaccharide -stimulated autophagic activation. Artesunate lost its anti-inflammatory function and ability to activate autophagy in TLR4-deficient macrophages, highlighting its connection with TLR4 activity. The study also found that artesunate blocked TRAF6–Beclin 1–PI3KC3 signaling and Beclin 1–PI3KC3 interactions. Recent research has demonstrated that artesunate suppresses the production of pro-inflammatory cytokines (i.e., TNF-α and IL-6) and protects mice against septic shock-induced death [104]. Although no direct link between artesunate and autophagy was investigated in the study, it was found that artesunate attenuates TLR4 and TLR9 expression, as well as NF-κB activation. It appears that although artesunate elicits therapeutic effects in various disease models, it can also promote opposing effects on regulating autophagic activity depending on the type of cell and animal model used. Based on mechanistic analyses [103,104], artesunate appears to target upstream signaling pathways that control autophagy rather than directly acting on core components of the autophagy machinery. This may partially explain the contradictory results observed following artesunate treatment. As different type of cells, tissues, and disease models rely on different upstream factors for the regulation of autophagic activity, artesunate could have distinctive effects on target proteins depending on the environment. Further studies that thoroughly exam these mechanisms could help to shed further light on the potential applications of artesunate as a therapeutic agent.

### 8.5. Trehalose

Trehalose is a natural disaccharide found in a diverse range of non-mammalian species that protects cells from oxidative stress [105], inhibits inflammation [106], ameliorates neurodegeneration [107,108], and induces autophagy [109,110,111,112,113]. It has been suggested that trehalose increases the activation of autophagy via an mTOR-independent pathway [112]. Observations suggest that the activation of autophagy by trehalose increases human rhinovirus replication in normal human primary airway epithelial cells [114]. Human rhinovirus is the most common virus responsible for acute respiratory diseases, including asthma [115]. Trehalose-induced autophagy downregulates IFN-λ1 expression and increases HRV-16 load, while the inhibition of autophagy by *atg5* knockdown results in the recovery of impaired-λ1 expression by trehalose and subsequently reduces HRV-16 load. On the other hand, trehalose has been reported to suppress human cytomegalovirus infection in diverse cell types [113]. Human cytomegalovirus can spread between organs via the bloodstream and cause disease in the developing fetus when viral load reaches sufficiently high levels [116]. In addition, human cytomegalovirus has been associated with various diseases including atherosclerosis [117,118] and cancer [119,120]. Trehalose extends the formation and number of autophagosomes as well as autolysosomes in infected human foreskin fibroblasts, with a study demonstrating that trehalose inhibits human cytomegalovirus replication in human foreskin fibroblasts, human aortic endothelial cells, and neural cells [113]. It has also been shown that the effect of autophagy induced by trehalose against herpesviruses is varied and appears to depend on the circumstances of infection conditions for at least two herpesviruses, human cytomegalovirus and varicella-zoster virus [121]. In addition, in a 2,4,6-trinitrobenzenesulfonic acid-induced intestinal inflammation mouse model, trehalose suppressed symptoms of colitis through autophagy activation. The administration of trehalose rescued intestinal damage and weight loss induced by 2,4,6-trinitrobenzenesulfonic acid. Trehalose attenuated the mRNA expression of pro-inflammatory cytokines, including *TNF-*α, *COX-2, IL-1β, IL-6*, and *IL-10*, and M1 markers, such as *CCR7, CD11c, iNOS,* and *CD86* in colon tissue. Trehalose reduced the protein expression of cytosolic BCL10, p-IκBα, and nuclear NF-κB [122]. These results suggest that trehalose may act as a promising agent for the treatment of colitis through autophagy activation. Acting as an activator of autophagy through an mTOR-independent manner can elicit benefits in certain circumstances, and trehalose may therefore be helpful in controlling autophagy and immunity. However, as the activation of autophagy does not always help to suppress viral infections, the potential application of trehalose as a therapeutic agent requires further understanding.

### 8.6. Vitamin D3

Vitamin D is traditionally known for its role in maintaining bone health and ability to prevent rickets, osteomalacia, osteoporosis, and hyperparathyroidism [123,124]. Results from recent studies suggest that vitamin D regulates both the innate and adaptive immune system, as well as autoimmunity [125,126,127,128]. In particular, the immuno-regulatory effect of 1a,25-dihydroxyvitamin D3 (1a,25-(OH)_2_D3), an active form of vitamin D that promotes the induction of autophagy, has been reported to provide protective effects against *M. tuberculosis* infection [129]. *M. tuberculosis* is typically attacked by macrophages and neutrophils that are part of the innate immune response [130]. 1a,25-(OH)_2_D3 triggers autophagic activation in human monocytic THP-1 cells and human primary monocytes through cathelicidin. *M. tuberculosis* infection can also be counteracted with the treatment of 1a,25-(OH)_2_D3, which induces the upregulation of *Beclin-1* and *Atg5* gene expression. Human cathelicidin appears to be required for the colocalization of mycobacteria and autophagosomes mediated by 1a,25-(OH)_2_D3. These observations suggest that vitamin D stimulates autophagy to induce innate immune responses against *M. tuberculosis* infection.

### 8.7. Baicalin

Baicalin is a flavonoid compound derived from the roots of *Scutellaria baicalensis* [131], and has been used for the treatment of various diseases including inflammation [132], psoriasis [133], and cancer [134]. Baicalin has also been shown to influence the immune system [131,135] and improve antibacterial defenses against *Staphylococcus aureus* by enhancing lysozyme (LYSO)-mediated bacteriostasis [136]. Baicalin has been observed to elicit antimycobacterial and anti-inflammatory effects via the induction of autophagy in macrophages [137]. Baicalin induces autophagy in RAW 264.7 cells, as evidenced by the upregulation of LC3-II and downregulation of p62. The activation of autophagy in such circumstances can be attributed to downregulation of the PI3K–Akt–mTOR pathway. Baicalin also suppresses *M. tuberculosis*-mediated NF-κB induction, which is important for full activation of the NLRP3 inflammasome. These findings suggest that baicalin may be a novel therapeutic candidate that can limit inflammation and enhance antimycobacterial activity via the induction of autophagy.

### 8.8. Ginsenosides

Ginsenosides are major bioactive compounds present in ginseng (*Panax ginseng*), which has been used as a traditional medicine for the treatment of numerous ailments including skin aging [138], obesity [139,140], inflammation [141,142], and cancer for centuries [143,144,145]. Furthermore, ginsenosides have been shown to possess various immuno-modulatory activities. The ginsenoside Rb1 has been reported to alleviate inflammation in atherosclerosis models via the induction of autophagy [146,147,148]. In order to examine the effect of ginsenoside Rb1 on atherosclerosis, apolipoprotein E (ApoE)−/− mice were treated for 8 weeks at 10 mg/kg body weight [148]. In the early stage of atherosclerosis, ginsenoside Rb1 promoted anti-atherosclerotic effects, reducing the production of inflammatory cytokines including TNF-α, IL-1β, and IL-6. It was demonstrated that ginsenoside Rb1 attenuates apoptosis and induces autophagy in the aorta of ApoE−/− mice, with a higher number of autophagosomes in the aorta area. Additionally, ginsenoside Rb1 increased LC3-II and Beclin 1 expression and reduced p62 expression in endothelial cells of the aorta. Similarly, in another study, treatment with ginsenoside Rb1 in ApoE−/− mice resulted in strengthened plaque stability and reduced lipid accumulation via the activation of autophagy in macrophages [147]. Rb1-mediated AMPK phosphorylation was the primary mechanism for activating autophagy in macrophages. These findings suggest that ginsenoside Rb1 counteracts the development of atherosclerosis by promoting autophagy in endothelial cells and macrophages.

Protopanaxadiol, another ginsenoside, has been reported to counter the progression of endometriosis (EMS) by inducing autophagy and enhancing NK cell cytotoxicity [149]. Endometriosis is believed to occur in around 5–15% of women of reproductive age and 20–50% of infertile women [150,151], with a low level of autophagic activity in ectopic endometrial stromal cells (eESCs) [152]. In one study [149], protopanaxadiol treatment increased autophagy, while upregulating progesterone receptor expression and reducing estrogen receptor α expression in eESCs, suggesting that protopanaxadiol may be a potential agent for the treatment of EMS as an autophagy activator. In addition, ginsenoside Rg1 has been reported to enhance Th1 and Th2 responses to hepatitis B infection via the TLR4 pathway [153] and stimulate the CD4+ immune response with an increase in IL-2 gene expression in murine splenocytes [154]. Whether this immune response elicited by Rg1 is autophagy-dependent or not remains unclear, although Rg1 has previously been reported to induce autophagy through the AMPK–mTOR pathway in RAW 246.7 macrophages [146].

### 8.9. Epigallocatechin-3-Ggallate (EGCG)

Epigallocatechin-3-Ggallate (EGCG) is a bioactive compound abundant in green tea and has been the subject of broad interest as a therapeutic agent for various applications, including inflammation [89,155], obesity [156], and cancer [157,158]. The effects of EGCG on autophagy appear to be diverse and dependent on the circumstances at hand. It has been demonstrated that EGCG induces the activation of pathways involved in autophagy including *ATG16L2, SNCA, TM9SF1, Pink1,* and *PIM-2* in resting and unloaded plantaris muscles. However, EGCG also partly inhibits the autophagy proteins Beclin 1 and LC3-II/LC3-I in the reloaded muscles of aged rats. Previous studies have shown that EGCG stimulates autophagy in steatosis [159] and human herpesvirus 8 (HHV8) models [160]. In the steatosis model, EGCG suppresses hepatosteatosis with improvements in autophagy biomarkers including autophagic activation in HepG2 cells, autophagosome formation, and the phosphorylation of AMPK, a key autophagy regulator [159]. EGCG exhibits protective effects against HHV8 in primary effusion lymphoma (PEL) cells [160]. In PEL cells, ROS induced by EGCG results in an increase in autophagic activation via the upregulation of Beclin 1 expression, leading to the inhibition of HHV8 replication. However, other researchers have reported EGCG inhibits autophagy in a liver injury model [161]. Hepatitis refers to several forms of liver disorder and can lead to cirrhosis, liver cancer, and finally death [162]. To investigate the effect of EGCG on hepatitis, inflammatory factors were analyzed in mice with hepatitis induced by concanavalin A (ConA). It was suggested that EGCG attenuates pathological damage by alleviating the levels of inflammatory cytokines, including TNF-α, IL-6, IFN-γ, and IL-1β. In addition, EGCG suppressed autophagic activation by blocking Bcl-2/E1B-19K interacting protein 3 (BNIP3), an autophagy stimulator, through IL-6–JAKs–STAT3 signaling. These observations collectively suggest that EGCG is a potential therapeutic candidate for the treatment of hepatitis, but the diverse roles of EGCG in activating or suppressing autophagy and its subsequent impact on the regulation of immunity requires further investigation.

### 8.10. Rapamycin

Rapamycin is a potent mTORC1 inhibitor that can induce the autophagy signaling pathway. Transmissible gastroenteritis virus (TGEV) is a coronavirus that naturally infect pigs. TGEV infection leads to an increase in the number of autophagosomes in host cells. TGEV infection triggers the autophagic response, and pharmacological or genetic inhibition of autophagy enhances TGEV infection. Treatment with rapamycin can increase autophagy in TGEV-infected cells and restrict TGEV replication [163]. Similarly, treatment with rapamycin was able to increase autophagic-flux in porcine intestinal cells and restrict porcine epidemic diarrhea virus infection [164]. These results demonstrate that mTOR-dependent autophagy activation by rapamycin can help to counteract viral infectivity.

On the contrary, multiple lines of studies have reported that activating autophagy using rapamycin can display supportive roles on viral replication [149,165,166]. For example, replication of the respiratory syncytial virus (RSV) was suppressed when key molecules of the autophagy pathway (e.g., ATG5, ATG7, and Beclin 1) were genetically silenced, whereas treatment with rapamycin led to an increase in RSV replication [149]. The study found that RSV induces autophagy through ROS generation and AMPK activation, and that the RSV infection-induced autophagy was able to block host cells from going into apoptosis, promoting the replication of RSV. As rapamycin and its analogues are currently being prescribed in clinic for other indications, they are good candidates for further development into antiviral therapeutics. However, rapamycin-mediated autophagy produces diverse outcomes toward controlling viral replication depending on the type of virus and host, suggesting that cautious approaches should be made when developing rapamycin for antiviral purposes.

Reports have suggested that autophagy impairment might enable the development of systemic lupus erythematosus. Lupus nephritis is a disease characterized by kidney inflammation and is one of the most serious manifestations of systemic lupus erythematosus. Studies have shown that the mTOR pathway is upregulated in lupus nephritis and treatment with rapamycin can help maintain normal renal function and reduce anti-dsDNA levels [167,168]. A recent study using lupus-prone MRL^lpr/lpr^ mice revealed that the inhibition of autophagy-aggravated podocyte damage, whereas administration with rapamycin relieved podocyte damage [169]. Rapamycin has also been shown to suppress lipopolysaccharide-induced lung inflammation by increasing autophagy and attenuating NLRP3-mediated IL-1β and IL-18 secretion [170]. Collectively, rapamycin-induced autophagy may contribute to the amelioration of several types of inflammatory diseases.

### 8.11. Peptides and Mycobacterium

#### 8.11.1. Peptides

Several peptides have been designed from a region of autophagy proteins to bind with pathogen driven factors and trigger autophagy. Nef is a HIV-1 virulence factor that interacts with Beclin 1 and is required for efficient viral replication. Through mapping the Nef-interacting domain of Beclin 1, the study developed a Tat–Beclin 1 peptide. The peptide binds to Nef and inhibits viral replication by inducing autophagy in vitro and in vivo. Importantly, administration of Tat–Beclin 1 reduced mortality of neonatal mice infected with chikungunya virus [171]. Another peptide derived from Beclin 1 BH3 domain has been designed to bind to the γ-herpesvirus68 Bcl-2 homolog, which downregulates autophagy and participates in viral reactivation. This peptide was shown to selectively abrogate suppression of autophagy mediated by γ-herpesvirus68 Bcl-2 homolog [172].

#### 8.11.2. Mycobacterium Indicus Pranii (MIP)

*M. indicus pranii* (*MIP)*, also known as *Mw*, is a non-pathogenic mycobacterium that has been reported to elicit anti-cancer effects in various malignancies including melanoma [173,174], breast, cervical, oral, lung, bladder, liver, and prostate cancer [175]. In addition, *MIP* may represent a promising immuno-modulator for the treatment of tuberculosis. In guinea pig models of tuberculosis [176], *MIP* enhanced bacterial killing by increasing the number of antigen-presenting cells (APCs) and lymphocytes in lung tissue infected with a low dose aerosol of *M. tuberculosis* H37Rv. In infected peritoneal macrophages [177], it was also demonstrated that *MIP* significantly upregulates TLR-4 signaling, including its downstream components, indicating that *MIP* enhances the host immune response against tuberculosis. Furthermore, *MIP* activates NF-κB, resulting in increased levels of pro-inflammatory cytokines and NO production, enhancing the immune response. *MIP* was also shown to be a potent inducer of autophagy for the clearance of *M. tuberculosis* in RAW 264.7 macrophages [178]. Both Rab5 (a marker for early phagosomes) and Rab7 (a marker present on late phagosomes), were suppressed by *M. tuberculosis,* but recovered when treated with *MIP*, although not in macrophages pre-treated with 3-MA (an autophagy inhibitor). These observations suggest that *MIP* relieves the inhibition of phagosome maturation by *M. tuberculosis*, and this occurs via the activation of autophagy. When LC3 expression was silenced by siRNA, *M. tuberculosis* clearance induced by *MIP* was abolished. These results demonstrate that *MIP* induces autophagy which promotes the clearance of *M. tuberculosis* in macrophages. Further studies are required to determine how *MIP* interacts with and regulates autophagy.

## 9. Conclusions

Recent studies have revealed the key contribution of autophagy in both innate and adaptive immunity. Through genetic and pharmacologic manipulation of autophagy genes, we have learned that the autophagy pathway is not only used for the degradation of self-constituents, but also can be used for the degradation of foreign invaders. Unsurprisingly, pathogens have devised a series of strategies to evade autophagy-mediated immune responses as well as hijack the host autophagy process for their own survival and replication. In addition to infectious diseases, activity of the autophagy pathway is critical in orchestrating inflammatory signaling in chronic and systemic diseases. 

**Figure 1:**
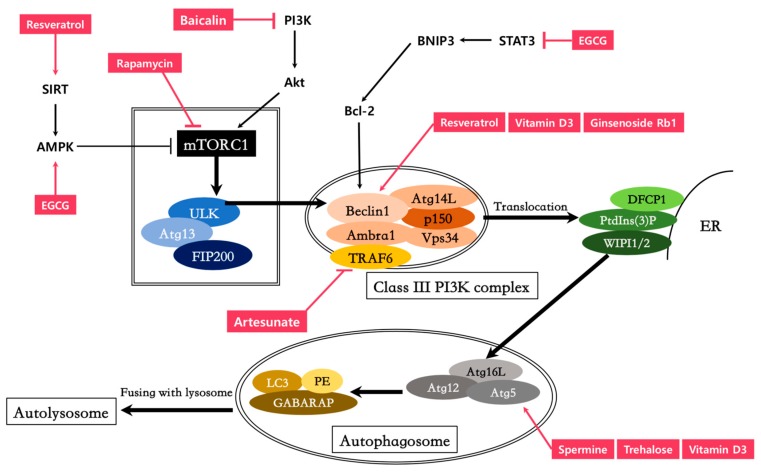
Compounds targeting the autophagy pathway for immuno-modulation. Akt, Protein Kinase B; SIRT, Sirtuins; TRAF, TNF Receptor Associated Factors; Atg, Autophagy related gene; FIP200, Family interacting protein of 200 kD; Ambra1, Activating molecule in Beclin 1-regulated autophagy; STAT3, signal transducer and activator of transcription 3; Bcl-2, B-cell lymphoma 2.

In light of the vital role autophagy plays in immune responses, the pathway has raised great expectations as a therapeutic target. Autophagy-modulating agents can have a major impact on controlling microbial infections and inflammatory responses (Figure 1 and Table 1). However, autophagy serves a dichotomous role in immunity, and its involvement in diverse biological processes further complicates its therapeutic application. The effects of autophagy during infection vary amongst cell types, stage of infection, and the type of pathogen. In spite of this, given the potent anti-inflammatory effects and crucial contribution to immunity, characterizing measures to delicately regulate autophagy might help strengthen the immune system. Future studies will have to focus on dissecting the molecular mechanism underlying the pathogen–host–autophagy interplay and investigate when autophagy inducers/inhibitors might exert proper immuno-modulatory effects. 

## Figures and Tables

**Table 1:** Immunity control and molecular mechanism of autophagy modulating compounds.

Compounds	Mechanism of Inducing Autophagy (Activation/Inhibition)	Related Immunity Model	References
Spermine	Increase ATG5 expression (Autophagy activation)	Liver inflammation	[68]
Spermidine	Unknown (Autophagy activation)	CD8(+) T cell response	[72]
Resveratrol	Increase SIRT3 expression & phosphorylation of AMPK (Autophagy activation)	Macrophage reactive oxygen species	[87]
	Increase SIRT3 expression & increase Beclin-1 expression (Autophagy activation)	Splenocyte viability	[88]
	Increase AMPK phosphorylation (Autophagy activation)	Peritoneal inflammatory injury	[89]
	Activation of cAMP-PRKA-AMPK-SIRT1 pathway (Autophagy activation)	Atherosclerosis	[90]
Artesunate	Inhibit TRAF6-Beclin 1-PI3KC3 (Autophagy inhibition)	Inflammation in peritoneal macrophages	[103]
Trehalose	Increase ATG5 expression (Autophagy activation)	Human rhinovirus infection (increase)	[114]
	Unknown (Autophagy activation)	Human cytomegalovirus infection (decrease)	[113]
	Unknown (Autophagy activation)	Colitis	[122]
Vitamin D3 (*1a,25-dihydroxyvitamin D3*)	Increase Beclin 1 & ATG5 expression (Autophagy activation)	*Mycobacterium tuberculosis* infection	[129]
Baicalin	Suppress PI3K-Akt-mTOR pathway (Autophagy activation)	*Mycobacterium tuberculosis* infection	[137]
Ginsenosides Rb1	Increase Beclin 1 expression (Autophagy activation)	Atherosclerosis	[147]
Protopanaxadiol	Unknown (Autophagy activation)	NK cell cytotoxicity in eESCs	[149]
Epigallocatechin-3-gallate (EGCG)	Increase phosphorylation of AMPK (Autophagy activation)	Hepatosteastosis	[159]
	Increase Beclin 1 (Autophagy activation)	Protection against Human herpesvirus 8	[160]
	Inhibit IL-6/JAKs/STAT3/BNIP3 pathway (Autophagy inhibition)	Liver injury	[161]
Rapamycin	Inhibit mTORC1 (Autophagy activation)	Coronavirus infection (decrease)	[163,164]
	Inhibit mTORC1 (Autophagy activation)	Respiratory syncytial virus & duck enteritis virus infection (increase)	[149,166]
	Inhibit mTORC1 (Autophagy activation)	Lupus nephritis	[167,169]
	Inhibit mTORC1 (Autophagy activation)	Acute lung injury	[170]
